# F63. COGNITIVE CORRELATES OF THE NEGATIVE SYMPTOMS EXPRESSIVE AND EXPERIENTIAL DEFICIT FACTORS IN PSYCHOSIS

**DOI:** 10.1093/schbul/sby017.594

**Published:** 2018-04-01

**Authors:** Serge Sevy, Anzalee Khan, Jean-Pierre Lindenmayer, Susan McGurk, Benedicto Paker, Isidora Ljuri, Mohan Parak, Abraham Goldring

**Affiliations:** 1 Manhattan Psychiatric Center; 2 New York University; 3 Boston University

## Abstract

**Background:**

Primary negative symptoms of schizophrenia contribute heavily to functional disability. Treatment of these symptoms continues to be a major unmet need, even when positive symptoms are controlled. Recent factor analyses of negative symptoms using the PANSS and other symptom assessments in patients with schizophrenia have identified two factors of negative symptoms: expressive and experiential deficits. These two factors most likely have very different clinical, neurocognitive and neurobiological correlates. This study examines the clinical and cognitive correlates associated with expressive and experiential deficits in a large cohort of patients with psychosis before and after computerized cognitive remediation.

**Methods:**

This is a secondary data analysis of subjects enrolled in a cognitive remediation program for 12 weeks. One hundred fifty-one subjects age 18 - 55 with a DSM IV-TR diagnosis of schizophrenia, schizoaffective disorder or bipolar disorder were enrolled. Assessments of demographic, psychopathology (PANSS), cognition (MCBB), and daily living skills (UPSA-Brief) were conducted at baseline and endpoint. Exploratory (EFA) and confirmatory (CFA) factor analyses of PANSS items as well as Pearson’s correlations between factors, demographics, MCCB, and UPSA-Brief scores were examined at baseline and endpoint.

**Results:**

EFA baseline PANSS data resulted in the five-factor model of the PANSS with seven items attributed to the Negative Symptom Factor (NSF; N1, blunted affect; N2, emotional withdrawal; N3, poor rapport; N4, passive social withdrawal; N6, lack of spontaneity and flow of conversation; G7, motor retardation; and G16, active social avoidance). CFA of the NSF revealed a two-factor model consisting of an Expressive Deficit (N1, N3, N6, G7), and an Experiential Deficit (N2, N4, and G16). Difference tests comparing the one-factor and two-factor models found that the two-factor model exhibited significantly better fit than the one-factor model (χ2 = 67.117, df = 1, p ≤ 0.001; CFI = 0.92; Tucker–Lewis index TLI = 0.91; root mean square error of approximation RMSEA = 0.040; and Goodness of Fit index GFI = 0.93). There were significant correlations between the Expressive Deficit factor score and cognition: TMT- A (r=-0.259, p=0.001), BACS Symbol coding (r=-0.287, p=0.001), Category Fluency (r=-0.342, p=0.001), Hopkins Verbal Learning Test – revised (HTLV-R) (r=-0.236, p=0.05), Letter Number Sequencing (r=-0.256, P=0.001), and NAB Mazes (r=-0.409, p=0.001). The Expressive Deficit factor was also significantly correlated with the neurocognitive domains of Processing Speed (r=-0.352, p=0.001) and Reasoning/Problem Solving (r=-0.338, p=0.001). There were no significant correlations between either factor and UPSA-Brief or the MCCB cognitive composite. There were no significant correlations for change from baseline to endpoint in negative symptoms.

**Discussion:**

Our results support the negative symptom two-factor model of Expressive Deficit and Experiential Deficit domains. Only the Expressive Deficit factor was associated with baseline deficits in Working Memory, Processing Speed, Reasoning/Problem Solving and Verbal Learning. The association of the Expressive Deficit factor with significant cognitive impairments supports a more profound neurobiological dysfunction in contrast to the Experiential Deficit factor and may represent an important treatment challenge. The relevance of these findings for the treatment of negative symptoms in schizophrenia will be discussed.

